# Current Landscape in the Management of Aortic Stenosis

**DOI:** 10.3390/jcm14103542

**Published:** 2025-05-19

**Authors:** Peng Liu, Hanzhe Wang, Shijie Wang, Yazheng Shan, Nianguo Dong, Yin Wang

**Affiliations:** Department of Cardiovascular Surgery, Union Hospital, Tongji Medical College, Huazhong University of Science and Technology, 1277 Jiefang Ave, Wuhan 430022, China; moon990310@hust.edu.cn (P.L.); m202376227@hust.edu.cn (H.W.);

**Keywords:** aortic valve, aortic stenosis, aortic valve replacement, prosthetic valve, non-invasive ultrasound therapy, pharmacological treatment

## Abstract

Aortic stenosis (AS) poses significant risks to patient survival and quality of life. The management of AS extends beyond restoring valve function to encompass lifelong disease management. While curative treatments exist, advancements in therapeutic approaches and prosthetic valve technology continue to evolve. This review synthesizes recent developments in AS treatment modalities, prosthetic valve innovations, and their clinical implications, delineating the current therapeutic landscape.

## 1. Introduction

Aortic stenosis (AS), the most prevalent valvular heart disease, arises from calcific degeneration, congenital bicuspid aortic valve, or rheumatic etiology. With aging populations, degenerative calcific AS prevalence rises sharply, affecting about 5% of individuals over 50 years [1]. The bicuspid aortic valve, present in 1–2% of the population, predisposes to accelerated calcification and hemodynamic abnormalities [2]. Rheumatic heart disease remains a major contributor to AS in developing nations, impacting 40.5 million individuals globally [1]. Contemporary AS management emphasizes minimally invasive techniques, reduced procedural risks, and lifelong patient-centered care. This review explores recent advancements in treatment modalities and prosthetic valves, highlighting their interplay in shaping the current therapeutic landscape.

## 2. Current Landscape

**Figure 1:**
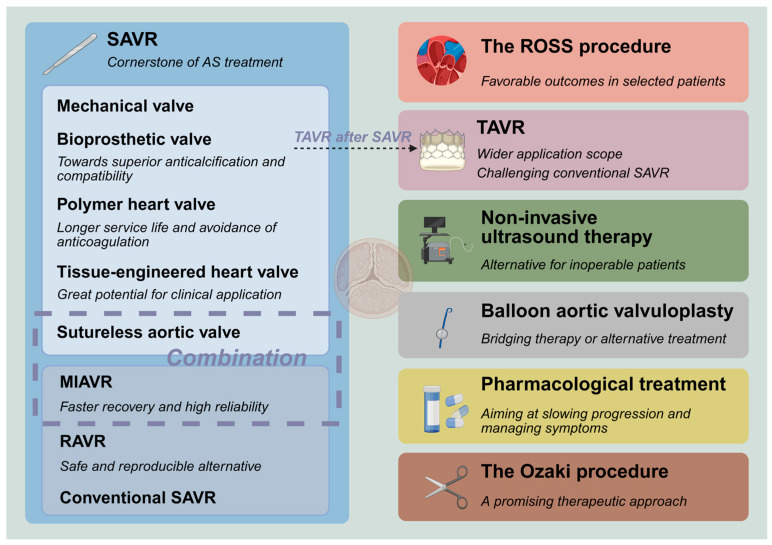
Current landscape in the management of aortic stenosis. Created with BioRender.com, with permission.

The various treatment modalities and prosthetic valves are interconnected and have formed the following landscape (Figure 1):

(I) Surgical aortic valve replacement (SAVR) is the cornerstone of AS treatment, and it serves as a salvage intervention when other treatment options fail;

(II) Bioprosthetic valves are evolving toward more superior anticalcification properties and compatibility with subsequent transcatheter aortic valve replacement (TAVR), which may lower the recommended age for use and bring about a higher quality of life;

(III) Polymer heart valves (PHV) have gradually emerged and achieved good early efficacy;

(IV) Tissue-engineered heart valves (TEHV) have great potential for clinical application;

(V) The progressive expansion of eligibility criteria for TAVR and the corresponding rise in procedural volumes have challenged the dominance of conventional SAVR;

(VI) Balloon aortic valvuloplasty (BAV) has undergone a shift in its role, functioning as a bridging therapy or an alternative treatment for high-risk patients;

(VII) Non-invasive ultrasound therapy (NIUT) has begun to be applied for calcific AS, demonstrating measurable improvements in valvular stenosis severity alongside a proven safety profile. Also, it may serve as a pretreatment before TAVR;

(VIII) Robot-assisted aortic valve replacement (RAVR) is a safe and reproducible alternative and can compete with TAVR;

(IX) Pharmacological treatment still rarely effectively slows down the progression of valve calcification. The anti-heart failure treatment also warrants clinical consideration;

(X) ROSS procedure (transplantation of auto-pulmonary valve) has started to return to people’s attention and yields favorable outcomes in some selected patient populations;

(XI) Minimally invasive aortic valve replacement (MIAVR) has become sufficiently mature, featuring excellent reliability and faster recovery. The application of sutureless valves in minimally invasive surgery can address the drawbacks of poor visibility and inconvenient manipulation;

(XII) The Ozaki procedure (autologous pericardial aortic valve reconstruction) demonstrates favorable short-term and mid-term outcomes, indicating its potential as a promising therapeutic approach.

## 3. Specific Description of the Current Landscape

### 3.1. SAVR—Cornerstone of the Treatment

SAVR remains the gold standard therapeutic approach for AS, characterized by its established safety profile and surgical maturity. The standardized operative protocol involves complete excision of the pathological aortic valve followed by prosthetic valve implantation under cardiopulmonary bypass with cardiac arrest, achieving definitive hemodynamic restoration. Nearly 100,000 cases of isolated SAVR were recorded in the Society of Thoracic Surgeons (STS) Adult Cardiac Surgery Database (ACSD) during 2018–2022, and the STS predicted risk of operative mortality was only 1.79% in 2022 [3,4]. While TAVR has expanding indications, there are still many cases suitable for SAVR, such as young age, inappropriate anatomy for TAVR (obvious dilatation of the valve annulus, low coronary ostium, severe aortic angulation, and unfeasible femoral access), severe concomitant valvular disease (severe aortic regurgitation, primary mitral regurgitation and tricuspid regurgitation), severe coronary artery disease, and so on [5]. As the cornerstone of AS management, SAVR demonstrates particular therapeutic superiority due to complete anatomical correction and can serve as a salvage intervention when other treatment options fail. Its irreplaceable role in comprehensive valve disease management continues to be validated through long-term clinical outcomes and evolving surgical refinements.

### 3.2. Advancement of Bioprosthetic Valves—Toward Superior Anticalcification and Compatibility

Bioprosthetic valves were introduced in the 1970s as an alternative to mechanical valves to avoid the need for long-term anticoagulant therapy. The advantages of bioprosthetic valves, including enhanced hemodynamic performance and decreased occurrences of thrombosis and bleeding events, have led to their widespread adoption in clinical settings. Consequently, bioprosthetic valves have supplanted mechanical valves as the preferred option for SAVR in developed nations [3,6]. However, the clinical utility of bioprosthetic valves is constrained by their limited long-term durability. A study involving 1387 patients who underwent SAVR with bioprosthetic valves showed that 181 patients (13%) had hemodynamic valve deterioration (HVD) caused by structural valve deterioration (SVD) during the first 5 years [7]. It can be inferred that patients under 40 years old who undergo SAVR with bioprosthetic valves are expected to undergo one or more reoperations over their lifetime, whereas approximately 60% to 75% of patients between 40 and 60 years old are likely to undergo reoperations [8]. However, valve-in-valve (VIV) TAVR can spare these patients the ordeal of undergoing thoracotomy again. It has demonstrated superior outcomes in terms of postoperative complications and early survival rates compared with redo-SAVR, but it has higher rates of myocardial infarction and is more likely to lead to severe patient–prosthesis mismatch [9].

In order to mitigate these limitations, the latest bioprosthetic valves offer enhanced hemodynamic performance, superior anticalcification properties, and a structure that accommodates future VIV TAVR. The INSPIRIS RESILIA aortic valve (Edwards Lifesciences, Irvine, CA, USA) was tested in the COMMENCE trial and demonstrated enhanced calcification resistance. This bioprosthetic valve is made with RESILIA tissue (Edwards Lifesciences, Irvine, CA, USA), which can completely block the free aldehyde groups in the leaflet tissue to prevent them from binding with calcium ions. It had stable hemodynamics and no evidence of SVD during the first 5 years [10]. The VFit technology (Edwards Lifesciences, Irvine, CA, USA) can make INSPIRIS RESILIA aortic valve obtain a controlled and predictable expansion during VIV TAVR. The PERIGON trial evaluated the safety and efficacy of the Avalus bioprosthesis (Medtronic, Minneapolis, MN, USA) and had no cases of SVD occur in both ≤65-year-old patients and >65-year-old patients at a 5-year follow-up [11]. Its mature AOA treatment (Medtronic, Minneapolis, MN, USA) uses α-amino oleic acid to prevent calcium ions from binding to tissues. The non-deformable polymer base of the Avalus bioprosthesis can benefit the future VIV TAVR, and the special structure can reduce the risk of coronary obstruction. It seems that the latest bioprosthetic valves demonstrate better performance compared to traditional bioprosthetic valves, but extended clinical surveillance and longitudinal data collection are still necessary.

### 3.3. Emergence of PHV—Longer Service Life and Avoidance of Anticoagulation

The PHV is designed to eliminate the requirement for prolonged anticoagulant therapy and to provide extended durability beyond bioprosthetic valves. At present, a variety of polymeric materials are under research [12]. The TRIA heart valve (Foldax, Salt Lake City, UT, USA) represents the inaugural PHV to progress into clinical evaluation. It was implanted through SAVR and has demonstrated favorable functional performance and improvement in the New York Heart Association (NYHA) functional class within one year after implantation [13]. Another milestone was achieved with the SIKELIA valve (MitrAssist Lifesciences, Shanghai, China), the first transcatheter PHV implanted in humans. It was designed to have a longer expected service life than the current transcatheter valves and obtained excellent one-year follow-up results [14]. Ongoing clinical trials are systematically evaluating the long-term durability and therapeutic efficacy of PHVs to establish their clinical value.

### 3.4. Development of TEHV—Great Potential for Clinical Application

To provide solutions for tissue creation and repair, Robert Langer and Joseph P. Vacanti introduced the concept of tissue engineering in 1993 [15]. TEHV represents a prominent application of this technology. These valves utilize either decellularized extracellular matrix or polymer scaffolds as their structural foundation and are implanted in patients in either cell-free form or pre-seeded with the patient’s own cells. They will undergo gradual remodeling to become functionally equivalent to native heart valves. Although no fully mature TEHV has yet been clinically approved, significant progress has been achieved over the past decade, and it has great potential for clinical application. A prospective observational study involving 69 patients evaluated the application of decellularized aortic valve homograft for AVR in pediatric and young adult populations [16]. The postoperative evaluation demonstrated extensive valvular recellularization and excellent valve function, suggesting that decellularized allograft heart valves may serve as a viable alternative to conventional prostheses for AVR in young patients. Current research should continue to explore optimal scaffold materials, cellular sources, and decellularization methods for TEHV, and large-scale clinical research remains essential to validate their clinical efficacy.

### 3.5. Wider Application Scope of TAVR—Challenging Conventional SAVR

TAVR has achieved remarkable advancements over the past two decades, commencing with Alain Cribier’s successful percutaneous implantation of an artificial valve to improve hemodynamics and relieve clinical symptoms in patients with severe AS in 2002 [17]. Procedural volume has demonstrated exponential growth in recent years. According to the data recorded in STS-ACSD, the volume of TAVR increased by 39% in 2020 compared to 2018 and by 66% in 2022 [4]. The evolution of TAVR has been validated through multiple comparative clinical trials, such as the PARTNER Trial, NOTION Trial, Evolut Low-Risk Trial, and other series. Unlike the initial indication limited to high surgical risk, the outcomes of these clinical trials demonstrated that TAVR, in middle-aged and elderly patients across the full surgical risk spectrum, is superior to or equivalent to surgical bioprosthetic valve replacement in terms of all-cause mortality, stroke or myocardial infarction, and bioprosthetic valve failure over the medium to long term follow-up [18,19,20,21]. These robust clinical outcomes have not only propelled the widespread adoption of TAVR but also challenged the dominance of conventional SAVRs. However, the utilization of TAVR in younger patients continues to be approached with caution. While clinically employed in this population, evidence showed inferior 5-year survival rates for TAVR versus SAVR in patients aged <60 years [22], highlighting the need for thorough risk–benefit assessment.

In addition, it should be noted that coronary artery obstruction, TAVR-related cerebral embolism, complicated anatomical structures in the bicuspid aortic valve, and vascular-related complications are still problems that TAVR needs to face. Therefore, in the subsequent research and development process, it should continue to improve the valve design, optimize the brain protection strategy, adjust the strategy of operation for the bicuspid aortic valve, and improve the delivery system.

### 3.6. The Changed Role of BAV—Bridging Therapy or Alternative Treatment

With the widespread adoption of TAVR, BAV has transitioned from a primary therapeutic option to a secondary role in the management of AS. It can currently serve as a bridge to permanent AVR, an adjunct to TAVR, a bridge to urgent or high-risk noncardiac surgery, a therapeutic choice for critically ill patients who are not suitable for TAVR, a palliative therapy, or an intervention for congenital AS [23]. But, patients with concomitant aortic aneurysm, moderate to severe aortic regurgitation, active endocarditis, vegetations, or left ventricular thrombus are not suitable candidates for BAV [24]. Notably, a meta-analysis suggested that TAVR with preimplantation balloon valvuloplasty had similar efficacy and safety to direct TAVR [25]. It highlights the need for evaluation of the necessity of preimplantation balloon valvuloplasty and further studies to obtain a definitive conclusion.

### 3.7. Clinical Application of NIUT—Alternative for Inoperable Patients

Although SAVR and TAVR represent established therapies for calcific AS, a subset of patients remains ineligible due to contraindications or procedural risks. This clinical issue underscores the urgent need for non-invasive intervention strategies capable of stabilizing disease progression and alleviating symptoms. The NIUT implemented by the Valvosoft device (Cardiawave, Levallois-Perret, Île-de-France, France) recently demonstrated therapeutic potential through clinical trials. The 6-month follow-up study involving 40 patients revealed favorable safety outcomes alongside objective improvements in hemodynamic parameters, including increased aortic valve area and reduced mean pressure gradient, accompanied by improved NYHA functional class and quality-of-life metrics [26]. It can serve as an alternative treatment for patients unsuitable for SAVR and TAVR and seems to be able to delay the progression of mild to moderate calcific AS. Additionally, preliminary evidence supports its utility as a pre-procedural intervention prior to TAVR, potentially mitigating complications such as prosthetic valve underexpansion and paravalvular leak [27].

### 3.8. RAVR—Safe and Reproducible Alternative

As mentioned above, TAVR has produced clinical outcomes comparable to SAVR across diverse AS patient cohorts, thereby challenging the dominance of conventional SAVR. RAVR, as an emerging branch of SAVR, may change this current situation. This fully robotic-assisted surgery has garnered increasing clinical adoption, and many medical institutions have gained experience with RAVR. It provides a clearer field of vision and enables more precise surgical maneuvers. Research involving 50 patients undergoing RAVR demonstrated satisfactory short-term safety and efficacy in low-surgical-risk populations, with perioperative mortality and morbidity rates showing non-inferiority, or even superiority, to TAVR [28]. In these 50 patients, all surgical times remained stable after the initial five procedures, demonstrating a clear learning curve effect. A propensity-matched analysis reported similar results. It showed that RAVR had a lower paravalvular leak rate and mortality in the first year after surgery in low and intermediate-surgical-risk patients compared with TAVR [29]. Notably, RAVR could also perform concomitant procedures like mitral repair, left atrial appendage obliteration, Cox–Maze ablation, and others safely, thereby expanding therapeutic capabilities beyond isolated valve replacement [28,29].

### 3.9. Exploration of Pharmacological Treatment—Aiming at Slowing Progression and Managing Symptoms

The pharmacological treatment of diseases has two principal objectives: first, to modify the natural history of the disease process, and second, to palliate clinical symptoms. Despite extensive research on the mechanism of calcific AS, which has significantly enhanced our comprehension of its pathogenesis, regrettably, at present, current pharmacological treatment has demonstrated limited efficacy in halting the progression of this disease. The clinical trials conducted on statins aimed at reducing low-density lipoprotein cholesterol (LDL-C) levels, as well as those targeting bone metabolism through the use of denosumab and alendronate or the supplementation of VitK2+ VitD, had yielded unsatisfactory outcomes in inhibiting the development of aortic valve calcification [30,31,32]. Notably, recent findings from ataciguat clinical trials revealed promising outcomes, as six-month treatment significantly attenuated calcification progression in patients with fibrocalcific AS, likely through modulation of soluble guanylate cyclase (sGC) signaling pathways [33]. Collectively, this research highlights the complexity of calcific AS pathogenesis and underscores the necessity for continued translational research to identify viable therapeutic targets.

AS may progress to heart failure (HF). Another objective of pharmacological treatment is to manage the symptoms associated with HF and maintain hemodynamic stability. Guideline-directed medical therapy (GDMT) for HF with reduced ejection fraction includes four medications [34]: (1) renin–angiotensin system inhibition with angiotensin receptor–neprilysin inhibitors (ARNI), angiotensin-converting enzyme inhibitors (ACEI), or angiotensin (II) receptor blockers (ARB); (2) beta-blockers; (3) mineralocorticoid receptor antagonists (MRA); and (4) sodium-glucose cotransporter 2 inhibitors(SGLT2i). Despite advancements in HF management, a critical knowledge gap persists regarding standardized anti-heart failure treatment specifically tailored to AS pathophysiology. Clinical practice underscores the inevitability of pharmacological intervention in most AS patients, emphasizing the urgent need for dedicated research to establish evidence-based treatment protocols. It is noteworthy that increased vascular stiffness may be one of the pivotal factors contributing to heart failure in patients with AS. Research has indicated that increased vascular stiffness is highly prevalent among patients with degenerative AS. Despite undergoing SAVR or TAVR, the increased vascular stiffness still elevates the risk of cardiovascular mortality and the incidence of heart failure [35]. This finding further highlights the critical importance of anti-HF therapy in postoperative management.

### 3.10. Resurgence of the ROSS Procedure—Favorable Outcomes in Selected Patients

The ROSS procedure, first reported by Donald Ross in 1967 [36], entails autologous pulmonary valve transplantation to the aortic position with concomitant replacement of the pulmonary valve using a homologous graft. Despite its theoretical advantages, this surgical strategy experienced limited adoption during earlier decades due to technical complexity and unresolved concerns regarding durability and hemodynamic performance. However, according to the data recorded in STS-ACSD, the annual volume of the ROSS procedure has increased from 68 cases in 2015 to 346 cases in 2022 [4]. This resurgence reflects growing clinical interest and accumulating evidence supporting its efficacy. A network meta-analysis compared the ROSS procedure with mechanical valve AVR and bioprosthetic valve AVR. It demonstrated superior outcomes in the ROSS procedure, with significantly lower all-cause mortality and long-term stroke rates, indicating improved long-term prognostic profiles [37]. The longest follow-up research for adults indicated that the ROSS procedure can provide long-term survival rates comparable to those of the general population, and the majority of patients (71.1%) are free from reintervention at 25 years [38]. These findings position the ROSS procedure as a viable alternative for SAVR, particularly in younger patients seeking durable valve solutions. While the technical complexity of the ROSS procedure remains higher than conventional SAVR approaches, accumulating procedural experience correlates with enhanced outcomes and safety profiles, suggesting a learning curve effect. Ongoing refinements in surgical techniques and perioperative management are expected to further optimize clinical outcomes and expand the procedural applicability within specialized centers.

### 3.11. Maturity of MIAVR and Sutureless Aortic Valve—Faster Recovery and High Reliability

Over the past two decades, MIAVR has evolved from an experimental concept to a well-established clinical surgery. The currently mainstream approaches for MIAVR include ministernotomy (MS) and right anterior minithoracotomy (RT), with other approaches, such as the right parasternal approach, also being utilized. Current evidence substantiates the advantages of MIAVR over conventional SAVR, demonstrating accelerated postoperative recovery, enhanced quality of life metrics, and superior patient satisfaction rates while maintaining comparable safety and efficacy profiles [39,40]. This clinical superiority aligns with patient preferences and has garnered increased attention from surgical teams. Comparative analyses between MS and RT approaches have yielded conflicting findings. An analysis involving 694 cases suggested that the operative time and recovery time in the RT cohort were shorter than those in the MS cohort [41]. But, research with a larger sample size reported the opposite result: that the MS cohort had reduced operative time and hospital stays, as well as improved early and long-term mortality [42]. For AVRs that require simultaneous handling of the aortic annulus, aortic sinus, or ascending aorta, the MS approach can provide a clearer and more direct surgical field to meet the needs of more complex operations [43,44]. Several studies compared the outcomes of MIAVR and TAVR and found similar safety and effectiveness. Notably, MIAVR cohorts exhibited a lower incidence of paravalvular leak but higher rates of acute kidney injury compared to TAVR [45,46,47,48].

The sutureless aortic valve, composed of a bioprosthetic valve and an anchored stent, facilitates rapid and precise implantation following the excision of the native valve without the necessity for suturing. The systematic reviews proved that sutureless aortic valve has satisfactory mid-term and long-term mortality, durability, and hemodynamic performance [49,50]. Its rapidly deployable structure makes it suitable for AVR with concomitant procedures, MIAVR, patients with fragile aortic valve annulus tissue, and high-risk patients who are not suitable for TAVR [51]. Comparative analyses suggested that the sutureless aortic valve can obviously reduce the rate of paravalvular leak compared to TAVR [52,53]. Furthermore, its integration into MIAVR has been shown to mitigate prolonged aortic cross-clamp and cardiopulmonary bypass times associated with limited surgical access. MIAVR with a sutureless aortic valve may emerge as a promising competitor to TAVR, but definitive validation of its efficacy necessitates rigorously designed randomized controlled trials to establish clinical equivalence and superiority claims. Another noteworthy concern is the relatively high pacemaker implantation rate associated with sutureless aortic valve [54,55]. A 2022 study demonstrated a significant reduction in pacemaker implantation rate following modification of the sizing strategy [56], underscoring the need for meticulous valve size evaluation and continued design optimization.

### 3.12. Promotion of the Ozaki Procedure—A Promising Therapeutic Approach

The Ozaki procedure, also known as autologous pericardial aortic valve reconstruction, was initially reported by Shigeyuki Ozaki in 2011 [57]. It is a technique that involves the customized reconstruction of the aortic valve using glutaraldehyde-treated autologous pericardium for various aortic valve diseases. The Ozaki procedure demonstrates favorable short-term and mid-term outcomes, offering good hemodynamics and quality of life without the need for anticoagulation therapy [58,59]. As such, it represents a highly promising therapeutic approach and holds promise for widespread adoption. Currently, more long-term follow-up outcomes are warranted to further evaluate the therapeutic efficacy and refine patient population selection for the Ozaki procedure.

## 4. Conclusions

The evolution of AS treatment methods typically progresses through three phases: curing the disease, halting its progression, and optimizing treatment strategies. Currently, AS treatment has advanced into the second and third phases. While SAVR remains the cornerstone, the expanding application of TAVR challenges its dominance. MIAVR and RAVR have emerged as promising alternatives, and the ROSS procedure is regaining attention. NIUT and pharmacological treatments are under active exploration. BAV still retains its unique role, and the Ozaki procedure holds great potential.

With regard to the selection between SAVR and TAVR, patients who are young, have unsuitable anatomical conditions for TAVR, suffer from severe concomitant valvular diseases, or have severe coronary artery disease are more appropriate candidates for SAVR [5]. Conversely, elderly patients are more suitable for TAVR. MIAVR is a highly appealing option for patients undergoing isolated aortic valve surgery. Although BAV and NIUT have more limited therapeutic efficacy compared to valve replacement, they can both serve as alternative therapeutic options for patients who are not suitable candidates for SAVR or TAVR. Both the Ross procedure and the Ozaki procedure possess their unique appeals. Each treatment modality has its own advantages and disadvantages, necessitating individualized patient selection based on factors such as age, life expectancy, comorbidities, aortic valve structure, lifestyle preferences, and medication compliance.

## Data Availability

Data availability is not applicable to this article, because no new data were published.

## Abbreviations

The following abbreviations are used in this manuscript:

ACEI	Angiotensin-converting Enzyme Inhibitors
ACSD	Adult Cardiac Surgery Database
ARB	Angiotensin (II) Receptor Blockers
ARNI	Angiotensin Receptor–Neprilysin Inhibitors
AS	Aortic Stenosis
BAV	Balloon Aortic Valvuloplasty
GDMT	Guideline-directed Medical Therapy
HF	Heart Failure
HVD	Hemodynamic Valve Deterioration
LDL-C	Low-density Lipoprotein Cholesterol
MIAVR	Minimally Invasive Aortic Valve Replacement
MRA	Mineralocorticoid Receptor Antagonists
NIUT	Non-invasive Ultrasound Therapy
NYHA	New York Heart Association
PHV	Polymer Heart Valves
RAVR	Robot-assisted Aortic Valve Replacement
SAVR	Surgical Aortic Valve Replacement
SGLT2i	Sodium–Glucose Cotransporter 2 Inhibitors
STS	Society of Thoracic Surgeons
SVD	Structural Valve Deterioration
TAVR	Transcatheter Aortic Valve Replacement
TEHV	Tissue-engineered Heart Valves
VIV	Valve-in-valve
